# The changing landscape of thyroid eye disease: current clinical advances and future outlook

**DOI:** 10.1038/s41433-024-02967-9

**Published:** 2024-02-19

**Authors:** Malik Moledina, Erika M. Damato, Vickie Lee

**Affiliations:** 1 Oculoplastics Service, Western Eye Hospital, Imperial College Healthcare NHS Trust, London, UK; 2 Department of Ophthalmology, Cambridge University Hospitals NHS Foundation Trust, Cambridge, UK

**Keywords:** Autoimmune diseases, Antibody therapy

## Abstract

**Aims:**

This review aims to provide an overview of the current understanding of TED and its pathophysiology. To describe the evidence base for current consensus treatment recommendations and newer biological therapies available as well as to present future therapeutic research.

**Methods:**

We reviewed and assessed the peer-reviewed literature placing particular emphasis on recent studies evaluating the pathophysiology of TED, landmark trials forming the basis of current management and recent clinical trials informing future therapeutics. Searched were made in MEDLINE Ovid, Embase Ovid, US National Institutes of Health Ongoing Trials Register and EU Clinical Trials Register. Keywords included: “Thyroid Eye Disease”, “Graves Orbitopathy”, “Thyroid Orbitopathy” and “Graves’ Ophthalmopathy”.

**Results and conclusions:**

The pathophysiology of TED involves a complex array of cellular and humoral based autoimmune dysfunction. Previous therapies have been broad-based acting as a blunt instrument on this mechanism with varying efficacy but often accompanied with a significant side effect profile. The recent development of targeted therapy, spearheaded by Teprotumumab has led to an array of treatments focusing on specific components of the molecular pathway optimising their impact whilst possibly minimising their side effect profile. Future challenges involve identifying the most effective target for each patient rather than any single agent being a panacea. Long-term safety profiles will require clarification as unintended immunological consequence downstream may become manifest as seen in other diseases. Finally, future novel therapeutics will entail significant expenditure and may lead to a divergence of available treatment modalities between healthcare systems due to funding disparities.

## Abstract (zh)

本文旨在对目前对甲状腺眼病 (TED) 及其病理生理学进行概述。阐述当前共识治疗建议和可用的新生物疗法的证据基础, 并介绍未来的治疗研究。

我们回顾并评估了同行评审的文献, 特别强调了评估TED病理生理学的最新研究、构成当前管理基础的里程碑式试验以及为未来治疗提供信息的最新临床试验。在MEDLINE Ovid、Embase Ovid, 美国国立卫生研究院正在进行的试验注册和欧盟临床试验注册中进行了检索。关键词包括: “甲状腺眼病”、“Graves眼眶病”、“甲状腺眼眶病”和“Graves眼病”。

TED的病理生理学涉及一系列复杂的细胞免疫和体液免疫障碍。既往治疗方法在该机制上起到了广泛的作用, 取得了不同程度的成功, 但往往伴随着严重的副作用。Teprotumumab介导的靶向治疗的发展带来了一系列针对分子通路特定治疗方法, 在优化疗效的同时可能将副作用降至最低。未来的挑战包括为每位患者确定最有效的靶点, 而不是将任何单一药物视为万能药物。对于靶向药物的下游有可能出现免疫反应, 因此需要明确长期安全性, 这一点在其他疾病中也有所表现。最后, 未来的新型疗法将耗费大量支出, 并可能由于资金差异而导致医疗系统间可用治疗模式的差异。

## Introduction

Thyroid Eye Disease (TED) presents a significant disease burden affecting 25–50% of all patients with Graves’ disease [1, 2]. Graves’ remains the commonest cause of hyperthyroidism with a population prevalence of 2% and an annual incidence of 20 cases/100,000 persons [1–3] Although the risk of visual loss is low (2–8%) it exacts a significant economic and psychosocial burden [4]. This is manifest in population studies showing TED patients having an increased suicide risk compared to Graves patients without TED [5]. Although the inflammatory phase of TED may be self-limiting, the disfigurement and diplopia frequently persists in moderate/severe disease, with an adverse impact on Quality of Life (QoL), unless addressed by rehabilitative surgery [6].

## Epidemiology

TED also manifests in clinically euthyroid patients and those with a background of chronic autoimmune hypothyroidism e.g Hashimoto’s thyroiditis [7]. The estimated incidence is 5/100000/year and an approximate population prevalence of 155/100,000 so TED has relevance to both the general ophthalmologist and endocrinologist [8, 9].

We aim to provide an overview of the current understanding of TED pathophysiology. We also aim to describe the evidence base for current treatment, newer biological therapies and future therapeutic research.

## Pathophysiology

**Fig. 1:**
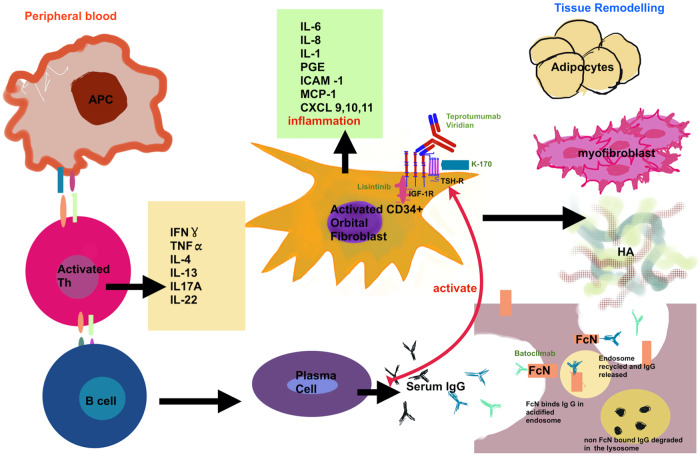
A pictorial representation of the pathogenesis of Thyroid Eye Disease. Depicts overexpression of TSH-Receptors (TSHR), on the orbital fibroblasts in TED patients. The activation of these, by IgG Thyroid Stimulating Antibody (TSAB) produced by plasma cells, results in the differentiation into adipocytes. Insulin-like growth factor-1 (IGF-1) and its receptors are also found on Orbital Fibroblasts Graves IgG Immunoglobulin, directed towards the IGF-1 receptor, results in activation of orbital fibroblast proliferation, cytokine secretion and hyaluronan synthesis. The two receptors IGFR-1 and TSHR, are co-localised on orbital fibroblasts, and are thought to have a synergistic relationship with a degree of cross-communication. Activation of the TSHR via the TSAB may also activate the IGF1-R intracellular cascade. The diagram shows the drugs Viridian, Teprotumumab and K-170 exerting their therapeutic effect by targeting these receptors. The diagram also shows Activated T-Cells and Orbital Fibroblasts secreting cytokines which encourages B-Cell maturation and subsequent secretion of TSAB by plasma cells resulting in further cellular recruitment, cytokine secretion and tissue expansion. Cellular structures are depicted which show the mechanism of antibody degradation and recycling. Batoclimab is shown to target the FcRn receptor, which is key in prolonging the half-life and preventing the degradation of IgG antibodies in circulation. Targeting this receptor blocks its function by competitively adhering to the IgG receptor site on FcRn and subsequently there is enhanced catabolism of IgG resulting in a drop in plasma levels.

The characteristic orbital inflammation and tissue expansion in TED has its pathophysiological basis in immunohistochemical studies [10]. These demonstrate an overexpression of TSH-Receptors (TSHR), on the orbital fibroblasts in TED patients [11]. The activation of these, either by excess hormone or Thyroid Stimulating Antibody (TSAB), results in the differentiation of orbital preadipocytes, a subgroup of orbital fibroblasts, into adipocytes, with consequential increase in orbital adipose tissue [12, 13]. Insulin-like growth factor-1 (IGF-1) and its receptors, overexpressed in T and B-Cells in Graves patients, is another critical player [14–16]. Graves IgG Immunoglobulin, directed towards the IGF-1 receptor, results in activation of orbital fibroblast proliferation, cytokine secretion and hyaluronan synthesis perpetuating inflammation and tissue expansion [17, 18]. The two receptors IGFR-1 and TSHR, are co-localised on orbital fibroblasts, and are thought to have a synergistic relationship with a degree of cross-communication [19, 20]. Activation of the TSHR via the TSAB may also activate the IGF1-R intracellular cascade [20]. In addition, orbital fibroblasts in TED patients may directly activate the adaptive immune response through their expression of CD40, a co-stimulatory protein [21]. The CD40 ligand (CD40L), found on CD4+ T-Cells, creates a CD40-CD40L bridge which actives orbital fibroblasts to release cytokines and immune mediators which promote fibrosis particularly late in the disease cycle [10, 22, 23]. Activated T-Cells and Orbital Fibroblasts secrete IL-6 which encourages B-Cell maturation and subsequent secretion of TSAB by plasma cells resulting in further cellular recruitment, cytokine secretion and tissue expansion [10, 23, 24] (Fig. 1).

## Clinical findings

The pathophysiological process of TED results in orbital inflammation, resulting in a constellation of signs and symptoms including grittiness, watering, ache and diplopia. Patients may develop ocular injection, periocular swelling, proptosis and strabismus.

Although sight-threatening disease is uncommon, milder disease may still impact Quality of Life QoL [25]. Several clinical manifestations of TED are explained by its aetiology described previously. Proptosis is hypothesised to result from intra-orbital volume expansion secondary to orbital muscle, orbital fat, or a combination of both [26]. Severe instances of intra-orbital volume expansion, which may impact between 2–8% of patients, might cause vision loss due to exposure keratopathy or compressive optic neuropathy [10, 27]. Inflammation and fibrosis of the upper-lid elevators, provide the typical symptom of lid retraction [10]. Gritty eyes, hyperaemia, periorbital oedema, chemosis, restrictive strabismus, and increased intraocular pressure are other prevalent signs and symptoms [28]. Clinical findings may be objectively assessed and scored by clinical activity scores, of which there are many.

## Clinical assessment scores

The clinical activity score (CAS) is long-established in Europe in assessing disease activity. Devised by Mouritis et al. [29], it is a 7-point (10-point post-presentation) binary scoring system that considers the symptoms and soft tissue signs indicative of anterior orbit inflammation. At presentation, CAS ≥ 3 (≥4/10 at subsequent visits) suggests active TED.

The European Group on Graves’ Orbitopathy (EUGOGO) recognises three categories of TED severity: mild, moderate-to-severe and sight threatening [7].

Alternative classification systems to measure disease severity include the modified NOSPECS classification. It is underutilised, due to its criteria involving a degree of subjectivity and its inability to measure activity [30]. The VISA Score, which measures both activity and severity, has been widely utilised across North America and adopted by International TED Society (ITEDS) for its studies. However, some clinicians have found it to be unwieldy and overtly complex in a non-academic setting [31].

There is no universal consensus on the assessment of TED-induced EOM dysmotility [32]. The Gorman scoring system provides a simple and quantifiable method of grading diplopia subjectively.

The GO-QOL (Graves Orbitopathy QoL) is the most widely adopted validated TED QoL outcome measure. This consists of a questionnaire divided into two subscales, the first evaluates the impact on visual function. The second analyses the psychosocial impact of the changed appearance from TED [33]. Studies have shown the scoring system to have good construct and cross-cultural reliability, validity and the ability to detect change [33, 34]. The questionnaire forms part of a series of outcome measures in TED trials and consensus guidelines have recommended regular use of these for assessment and monitoring of patients within a clinical setting [34].

## Early recognition and diagnosis

Prompt achievement of euthyroidism underpins TED management and a multi-disciplinary approach is recommended as optimal [25].

In 2009, renown global experts convened in Amsterdam to produce a series of objectives and five-year targets related to optimising the care and prevention of TED: the Amsterdam Declaration [35]. In conjunction with TEAMeD, the UK implementation taskforce, work began on a series of initiatives to realise these ambitions [35, 36].

## Current management

One such initiative is a guideline detailing a systematic, stepwise and structured approach in the management of TED, outlined by EUGOGO and is widely adopted in the UK and Europe [7]. The guideline structures the management of TED based on disease activity and severity [25]. Severity of disease is measured as per the EUGOGO’s own classification as mild, moderate-to-severe and sight threatening disease. It utilises measurements of lid retraction, exophthalmos, soft-tissue involvement, diplopia and the presence or absence of dysthyroid optic neuropathy and/or corneal breakdown [7]. Patients presenting with active disease, which is moderate-severe or sight threatening, are treated with high dose systemic glucocorticoids as first-line treatment in conjunction with Mycophenolate Sodium. This is outlined in the 2021 EUGOGO guidelines, with the emphasis that both therapeutics should be started concomitantly for greatest effect. Non-responders may require urgent orbital decompression surgery if sight threatening disease persists and second-line immunosuppressive agents in patients with moderate-severe disease [7]. It must be emphasised that the second-line immunosuppressive agents are most effective if started early in the disease course due to a delayed onset of action [11].

For inactive disease in the moderate/severe group with significant impairment of QoL, rehabilitation surgery (orbital decompression eyelid surgery to address the proptosis and eyelid retraction and strabismus surgery for intractable diplopia) remains the mainstay to alleviate residual dysfunction and disfigurement.

In our evaluation of the current evidence, we will place emphasis on the therapeutic efficacy: reduction in manifestations of inflammation (reduction in CAS), reversal of proptosis and improvement in ocular-motility as well as patient reported outcomes (GOQOL). Many of these features make up the objective EUGOGO composite index, which is a clinician reported outcome measure utilised as an objective criterion to determine patient response to treatment of moderate-severe active TED [7, 37].

## Glucocorticoids

Glucocorticoids (GC) have been integral to first-line immunosuppression in TED. They are most impactful when instituted early in the disease process, during the active phase on Rundle’s curve. The pharmacological basis of GC impact on the synthesis of glycosaminoglycans and the recruitment of pro-inflammatory mediators such as monocytes, macrophages, T and B-Cells [38]. GCs also promote anti-inflammatory and suppresses pro-inflammatory cytokines [38, 39]. In one small randomised trial (RCT) comparing Intravenous Methylprednisolone (IVMP) to placebo, 83% of patients with TED had a response to IVMP, when evaluating inflammatory changes and ocular-motility, versus 11% who received placebo [40]. Of those patients with ocular-motility dysfunction before the trial, 50% on IVMP responded (improvement of ≥8° in any direction) compared to 11% on placebo (*P* = 0.095). In the IVMP group, all patients achieved a reduction of CAS (≥2) compared to 33.3% in placebo (*P* = 0.01). Improvements in proptosis were less convincing with a reduction in 40% of IVMP patients (≥2 mm) versus 29% on placebo (*P* = 0.68). No serious adverse events (AE) were noted for either group.

IVMP has been shown in several RCTs and in a meta-analysis to be more efficacious than oral daily prednisolone at a cumulative dose of 4 g, with some studies reporting a 26% difference in response rate [41–44]. Furthermore, one meta-analysis found that patients being treated with oral GC had a higher rate of steroid induced AE relative to the IVMP group; predominantly weight gain, Cushingoid features and hypertension. The same meta-analysis, evaluating four trials, found statistically significant (ss) superiority of IVMP compared to oral corticosteroids in reducing CAS. However, no difference was found when evaluating VA, diplopia and proptosis [43].

There is heterogeneity on the ideal dosage of IVMP and protocols vary on several factors including disease severity [7, 38]. What is clear is that the steroid doses required for treatment are high, and the risk of treatment is justified when the clinical presentation is at least moderate and/or the patient’s QoL is significantly impaired. The current EUGOGO regimen involves six infusions of 500 mg IVMP followed by a further six weeks of 250 mg in moderate-severe disease; a cumulative dose of 4.5 g per cycle which is well tolerated [7, 44, 45]. For sight-threatening disease, higher doses up to a cumulative 7.5 g per cycle has been advocated due to short term advantages over lower doses [7, 46]. Safety concerns have been identified in cumulative doses of >8 g of Methylprednisolone per cycle. In one study, of the seven fatal cases that occurred, all but one case had a cumulative dose of >8 g of IVGC so a customarily cumulative doses of >8 g are not recommended. Patients undergoing IVGC should be monitored for AE including arrythmias, electrolyte and liver abnormalities, hyperglycaemia, hypertension, infection and provided with adequate gastric and bone protection [47].

## Second-line non-steroidal immunosuppressive treatment (NSIT)

The treatment strategy for NSIT can be viewed through the lens of either disease control or steroid sparing. Therefore, immunosuppression that achieves equivalence to glucocorticoids may still be considered beneficial by avoiding the myriad of steroid related AE. However, in order to be effective, most immunosuppression needs to be initiated early in the disease to be given time to achieve a therapeutic level. A patient may be mistakenly thought to be resistant to a particular agent whilst in reality treatment had been initiated to late in the disease cycle. These principles should be considered when evaluating and instituting NSIT.

In most scenarios, NSIT falls within the purview of failed glucocorticoid treatment or relapsed TED in moderate-severe active disease [7]. Published studies have demonstrated immunosuppressive treatment being effective in only 50–80% of cases and may not lead to complete resolution of activity [48]. Below we ascertain the evidence base for several options recommended by the EUGOGO consensus [7].

## Azathioprine

Azathioprine has been utilised in the management of organ transplantation and other chronic inflammatory disorders such as Rheumatoid Arthritis (RA). It mediates its effects via inhibiting purine synthesis thus impacting cellular replication within the bone marrow [49]. Azathioprine has not been utilised as a single agent in TED. A small prospective study performed by Perros et al., found Azathioprine did not influence any ophthalmic parameters measured including, exophthalmometer readings, palpebral aperture and visual acuity, relative to the control group [50]. The CIRTED double-blinded RCT found on post hoc analysis, patients treated with Azathioprine and oral glucocorticoids had better outcomes than those treated with oral glucocorticoids alone, as judged by the Binary Clinical Composite Outcome Measure (BCCOM). This was only the case when Azathioprine was continued for a minimum of 24 weeks. As steroid treatment was withdrawn at 24 weeks, Azathioprine is thought to mediate its effect by reducing the risk of relapse following steroid withdrawal. Better outcomes were defined as per BCCOM. The major criteria consisting of: ≥1 improvement in diplopia score, >8° of ductions in any direction and a reduction of in proptosis (≥2 mm). However, in a post-hoc analysis of the intention-to-treat patient cohort, Azathioprine did not improve the CAS score at 48 weeks. Nevertheless, the study did have limitations including the high dropout rate due to drug related AE [51]. The main safety profile concern, with the utilisation of Azathioprine, was the higher risk of deranged blood tests or related side effects compared to placebo (*p* = 0.00057). Several patients required rescue treatment including radiotherapy and corticosteroids. There was an absence of improvement in the GOQOL in any of the treatment cohorts [51].

A three-year follow up study of the original CIRTED trial, encompassing 54% of patients originally randomised with good distribution found no long-term improvement in the BCCOM score when comparing Azathioprine versus placebo. Furthermore, there was no difference observed in the EUGOGO severity score nor any improvement in the long term GoQoL at the three-year mark when evaluating Azathioprine versus placebo [52].

Due to a limited evidence base the impact of Azathioprine treatment in TED remains unclear. Despite this, the drug’s potential benefit of reducing the risk of relapse following steroid taper maintains its use in TED management.

## Cyclosporine

Cyclosporine is a calcineurin inhibitor, impacting the production IL-2 which plays a critical role in the activation of T-cells; a key mediator in the pathogenesis of TED [53]. Two small RCTs evaluated the use of Cyclosporine A in TED. The first study evaluated the efficacy of cyclosporine compared to prednisolone and found a 39% difference in response rate, measured via an improvement in VA, proptosis and a decrease in eye-muscle enlargement, in favour of prednisolone. However, when used in combination, 60% of patients who did not respond to either drug as a monotherapy, improved [54]. The second study evaluated the benefits of combination therapy, cyclosporine and prednisolone, versus prednisolone alone. It found combination therapy to be more effective, evaluated through a structured activity score composed of objective, subjective and radiological parameters, than prednisolone as monotherapy [55]. Cyclosporine has a propensity to cause serious side effects with the drug’s narrow therapeutic index requiring careful and regular monitoring. Despite this, studies have shown the drug to be better tolerated than prednisolone with a fast onset-of-action [54, 56].

## Mycophenolate

In the EUGOGO guideline, Mycophenolate is utilised as an adjunct to systemic glucocorticoids rather than a second-line modality [7]. It is thought to act by suppressing the actions of both T and B-Cells, subsequently impacting antibody production [39]. It also has a role in limiting the recruitment of cells that propagate inflammation, such as monocytes and lymphocytes, via inhibiting the expression of glycoproteins and adhesion molecules [57]. The drug has anti-fibrotic properties in lung and renal disease which may have applicability in TED [58, 59].

Mycophenolate is available as one of two formulations: Mycophenolate Mofetil (MMF) or Sodium (MS) which is enteric-coated [60]. Despite this, a recent systematic review has shown no difference in GI related QoL or AE between the two formulations [61]. Furthermore, it should be noted that 0.72 g of Mycophenolate Sodium has an equivalence of 1 g of Mofetil [7].

The MINGO study, an observer-masked multicentre randomised trial, recruited patients who had active moderate-to-severe TED. These patients were randomised to receive either IVMP (0.5 g weekly for 6 weeks then 0.25 g for a further six weeks) alone or in combination with MS (360 mg twice daily for 24 weeks). Response rate was defined as per a composite ophthalmic index consisting of several measures including CAS, diplopia, motility, lid width and swelling. A response was defined as an improvement in ≥2 of these measures without deterioration or relapse in others. The study was unable to show a significant difference, in response rate, between groups at 12 weeks. This is to be expected, considering that glucocorticoids are likely to be the predominant suppressor of inflammation at this stage. The effect of MMF is more pronounced after 12 weeks following the cessation of steroids and particularly when started early in active disease. The MINGO study was able to show a higher response rate of MMF, in a post-hoc analysis, with combination treatment compared to monotherapy at 24 weeks (71 versus 53% OR: 2.16. 1.09–4.25 *p* = 0.026) and 36 weeks (67 versus 46% OR 2.44, 1.23–4.82, *p* = 0.011) respectively [62]. This study did not show an increase in frequency of AE in combination therapy relative to monotherapy. This corroborates real-world-data which shows the relatively benign safety profile of Mycophenolate comparable to IVMP [62, 63].

One RCT showing Mycophenolate having a ss superior response rate compared to glucocorticoids at 24 weeks in patients with moderate-to-severe active TED, has been withdrawn [64]. This undermines the evidence base for the EUGOGO consensus recommending dual first line immunosuppression of IVMP with Mycophenolate [7, 64]. It remains to be seen whether this recommendation continues in the next iteration.

## Sirolimus

Sirolimus (Rapamycin), is an immunosuppressive agent which acts by modulating lymphocyte sensitivity to cytokines, predominantly IL-2, via the mTOR pathway. It also acts on myofibroblasts, and thus displays anti-fibrotic properties in renal and pulmonary transplant patients [65–67] but has a significant side effect profile and requires careful monitoring. A small observational study performed by Lanzolla et al. compared the effect of sirolimus versus IVMP, in patients who had active moderate-to-severe disease requiring second-line treatment. The sirolimus group had a superior response at 86.6% compared to 26.6% (*p* = 0.0026) in the IVMP group at 24 weeks. with an improved QoL(GO-QoL) Score, Diplopia (63.6 vs 23% *p* = 0.052), Proptosis (80 vs 13.3% *p* = 0.0011) and CAS (86.6 vs 33.3% *p* = 0.0062). In the trial Sirolimus was well tolerated with no serious events requiring a discontinuation or dose reduction [68]. This study’s limitations include a small sample size (*n* = 30) and the observational non-randomised study design. The conclusions may not be generalisable to treatment naive patients considering the cohort selected had either failed Methylprednisolone treatment in the first instance or it was contraindicated. A phase II RCT is underway, utilising sirolimus as a first-line agent, to address these limitations (NCT04598815) [68].

## Orbital radiotherapy

Orbital Radiotherapy (OR) utilises ionizing radiation that results in double-stranded DNA breaks inducing apoptotic cell death, particularly in susceptible T & B lymphocytes, macrophages and orbital fibroblasts [69, 70]. OR also modulates the nitric oxide pathway that has a role in inducing and propagating orbital inflammation [70].

There are several RCTs evaluating the therapeutic effect of OR on TED with contradictory findings. A meta-analysis reviewing 18 studies of which 10 were randomised and 8 were cohort studies concluded that OR is efficacious in the treatment of TED and has a greater impact when combined with glucocorticoids [71]. However, a second meta-analysis evaluating 33 RCTs concluded that the efficacy of OR monotherapy is of indeterminate benefit but combining OR with corticosteroids was more efficacious than monotherapy of either alone [43]. Therefore, the evidence base tends to be unclear but there is a tentative understanding that OR with oral corticosteroids is likely effective. OR is likely to be most effective in active disease, after a lag period allowing the ionizing radiation to sufficiently produce the double-stranded DNA breaks to induce apoptotic cell death. It is possible that studies, without a sufficient duration of follow up or without a large enough cohort of active patients, may be unable to show the potential benefits of OR in TED patients.

There is no consensus on the exact regimen of OR but a dose of 20 Gy divided over a two-week period (10 daily doses) is widely used [72]. The treatment is not advocated in those with significant diabetic retinopathy or hypertension as the treatment risks disease progression. Younger patients have a marginally increased risk of cancer when treated [73].

## Methotrexate

Methotrexate acts through its role as a folate antagonist [74]. It restricts the synthesis of de-novo pyrimidines and purines by inhibiting the enzymes thymidylate synthase (TYMS) and Dihydrofolate reductase (DHFR) [74, 75] This in turn inhibits the formation of DNA, RNA and proteins which impacts upon the proliferation of inflammatory cells including, T-helper and B-Cells critical in mediating the inflammatory response in TED [74, 75].

There is a paucity of RCTs evaluating the use of Methotrexate in TED. One small non-randomised study evaluated the use of Methotrexate in 36 consecutive patients as an alternative to GC treatment. Patients in this study had previously been treated with IVGC, followed by an oral taper but were stopped due to an inability to tolerate the side effects. Patients were initiated on a dose of either 7.5 mg or 10 mg weekly for a year dependent on patient weight. CAS, VA, Ocular-Motility, Exophthalmos and lid position were evaluated at 3,6 and 12 months which was then correlated with baseline readings. The study found a ss improvement in CAS at 3,6 and 12 months compared to an average baseline CAS of 4. Improvements were found at six and 12 months in ocular-motility. No improvements were found at any time point in VA, exophthalmos or lid position [76].

Two further retrospective studies evaluated the use of Methotrexate as an adjuvant to corticosteroids [77, 78]. The first study performed by Rivera-Grana et al., reviewed 14 patients who were treated with oral prednisolone (average of 32 mg/day). All patients included were unable to discontinue oral prednisolone without recurrence. Of the nine patients who continued on Methotrexate, were all able to discontinue prednisolone after an average duration of 7.5 months. The authors also noticed an improvement in visual acuity and partial improvement in ocular-motility but the results were not statistically significant [77].

A second retrospective study by Yong et al., compared Methotrexate with and without IVMP in a cohort of 72 patients with sight-threatening TED. The VISA score was the primary outcome measure evaluated at 0, 3, 6, 12 and 18 months. Both groups of patients had over 80% of active subjects, with a similar duration of active TED. The study found in those patients, who underwent combination treatment, a ss improvement in VA of >2 lines on Snellen and VISA inflammatory score at three months but at no other time points. There was no difference in proptosis or restrictive myopathy [78].

In all studies Methotrexate appeared to be well tolerated without any significant adverse events reported. Three patients who received combination treatment in the study performed by Yong et al. had worsening of liver function tests but these subsequently resolved on cessation. A sizeable proportion of the patient cohort investigated by Rivera-Grana et al. reported Fatigue (29%), Nausea (21%) and Hair loss (14%) [76–78].

Methotrexate appears to show some promising results particularly in the use of sight-threatening TED in combination with glucocorticoids. However, the lack of good quality RCTs limits our understanding as to the magnitude of its effect in TED.

## Non-immunosuppressive adjunct therapies

### Statins

Statins act via the competitive inhibition of the enzyme HMG-CoA reductase which is critical in the pathway for cholesterol synthesis [79]. Studies shown that statins also possess anti-inflammatory properties which are mediated through the apoptosis of cells that propagate inflammation [80, 81].

Two large longitudinal cohort studies, one in the US and the other in Sweden, found a significantly decreased hazard of developing TED in patients taking statins not seen in other cholesterol lowering treatments [82, 83]. One small RCT study randomised patients with active moderate-to-severe TED and hypercholesterolemia to either IVMP and statins or IVMP alone found the statin arm were more likely to respond to treatment than those in the non-statin arm (51% compared to 28% *p* = 0.042). Response was defined as per a combined composite evaluation of a number of variables including VA, CAS, diplopia, lid aperture and exophthalmos. However, statin’s beneficial effect on patients with normal cholesterol levels has not been quantified [84].

### Selenium

Selenium exists in high concentrations within the thyroid gland and confers a protective anti-oxidative benefit on thyrocytes during the synthesis of thyroid hormones which are elevated in Graves Hyperthyrodism [85, 86].

A landmark double-blinded, RCT found patients who were given sodium selenite (200 µg daily for six months) had significantly better ophthalmic outcomes, reduced rate of progression and higher GO-QoL scores compared to placebo or pentoxifylline [87]. These effects persisted for six months following sodium selenite withdrawal. A significant reduction in CAS at six and 12 months was observed compared to placebo. However, most improvements were observed in soft tissue changes and lid aperture rather than proptosis or motility. Furthermore, no AE were identified, in the sodium selenite group unlike pentoxifylline where patients suffered from gastrointestinal problems. However, the study did not investigate whether the patients recruited were in-fact sodium selenite deplete on recruitment and how treatment impacted on blood levels. Selenium is now recommended to all patients with mild-moderate TED, for a period of six months, to reduce the likelihood of progression. However, the original study group consisted of patients only with mild TED [87].

### Biologics

The use of monoclonal antibodies (MAB), is established in the treatment of other autoimmune inflammatory conditions, although it is a new advancement in TED. The evidence base behind the efficacy of biologics is developing rapidly. The EUGOGO guidelines make reference to three specific treatments as second-line agents in the management of moderate-to-severe active disease: Tocilizumab, Rituximab and Teprotumumab [7]. We will explore each below.

### Tocilizumab

Tocilizumab is a MAB, directed against interleukin-6 receptors (IL-6R) [88]. It is licensed for the treatment of Castleman’s disease, RA and Giant Cell Arteritis [89, 90]. The drug acts via the inhibition of Interleukin 6 (IL-6), which is a key mediator of inflammation via T-cell activation and the expression of pro-inflammatory proteins such as C-Reactive Protein. IL-6 also has a role in the maturation and differentiation of B-cells resulting in antibody production, and volume expansion within the orbit [91].

A Cochrane review evaluating the use of Tocilizumab in TED was unable to come to a conclusion due to a lack of high quality RCTs [92]. Nevertheless, a small double-blinded RCT performed by Perez-Moreiras et al. evaluated patients with active moderate-to-severe glucocorticoid resistant TED [93]. Glucocorticoid resistant TED was defined as previous treatment with pulsed IVMP without improvement to GO. Total IV steroid dose, in each patient, ranged from 1.5–7.5 g. Patients were given either an infusion of Tocilizumab or placebo administered at weeks 0, 4, 8, and 12. At week 16 patients in the Tocilizumab group showed greater reductions in CAS (86% CAS < 3) versus placebo (35% *P* < 0.005). GoQoL was found to significantly improve at week 16 in the Tocilizumab compared to placebo There were also improvements in exophthalmos, in the Tocilizumab group compared to placebo. However, the effects were not as pronounced with a median change of −1.5 mm. At week 40, changes in exophthalmos and diplopia were not ss between the groups. Other uncontrolled studies have shown larger changes in exophthalmos with one study reporting a mean reduction of −3.92 ± 1.54 mm in 72% of patients [94].

In the Perez-Moreiras study, Tocilizumab was well tolerated though there were a higher number of infections in the treatment arm with one patient developing acute pyelonephritis [93]. Other uncontrolled studies seem to validate the effectiveness of Tocilizumab, particularly in patients with steroid resistant TED [95–97].

A multi-centre observational study performed by Sánchez-Bilbao, whose aim was to evaluate the efficacy and safety of Tocilizumab in 48 TED patients unresponsive to conventional therapy [96]. The study’s primary outcome variables consisted of BCVA, IOP and CAS. Secondary outcome measures included proptosis. The majority of patients were given Tocilizumab alone (*n* = 45) at a dose of 8 mg/kg IV monthly or 162 mg subcutaneously weekly. A minority also received a conventional immunosuppressive agent (*n* = 3) in addition to Tocilizumab. At one year, all primary outcome variables showed a ss improvement. At baseline CAS was 4.64 ± 1.5 which reduced to 1.05 ± 1.27; (*p* = 0.0001) at one year following treatment. Following a mean follow up of 16.1 ± 2.1 months, inactive disease was achieved in 92.6% of eyes. Secondary outcome measures such as proptosis also showed promise with a 23% reduction of patients experiencing a > 2 mm increase in proptosis compared to baseline at 12 months (*p* < 0.05) [96].

The lack of RCTs evaluating the effectiveness of Tocilizumab in steroid naive patients, limits the quality of evidence available to evaluate Tocilizumab’s true efficacy. To address this deficiency, we anticipate the outcome of ongoing trials [56].

### Rituximab

Rituximab, a monoclonal antibody against CD20, is well established in the treatment for a variety of autoimmune diseases. It acts via CD20 receptor expressed on B-Cells, resulting in B-Cell depletion [98].

Two single-centre RCTs have shown conflicting results in the response to treatment with Rituximab. A prospective randomised double-blinded study from the Mayo Clinic, with 21 subjects given Rituximab (two doses of 1000 mg each two weeks apart) or placebo (saline), found no difference in CAS scores at 24 or 41-weeks weeks (31% Rituximab and 25% placebo, *P* = 0.75). There were no statistically significant changes in proptosis, SF-12 and diplopia scores [99]. One subject in the Mayo study progressed onto dysthyroid optic neuropathy and another vasculitis [99].

A second double-blinded randomised trial from Milan compared 32 subjects randomised to IVMP or Rituximab (2000 or 500 mg) in a similar severity cohort and found 100% disease inactivation in the Rituximab cohort (CAS < 3) compared to 69% in IVMP (*P* = 0.043) at 24 weeks. The average CAS by 24 weeks, had declined to 2.3 ± 0.5 and 0.6 ± 3 in the IVMP and Rituximab groups respectively (*P* < 0.006). In addition, there were no disease re-activation in the Rituximab compared to five subjects in the IVMP cohort by 54 weeks. The Rituximab cohort also showed significant ocular-motility improvement as a secondary outcome measure at 52 weeks, although there was no significant improvement in proptosis or diplopia scoring. Ss improvements were noted at 52 weeks on both the GoQoL appearance and visual functioning scales (*P* = 0.027 and *P* = 0.01 respectively) [100]. Two Rituximab subjects developed a cytokine-release syndrome which was responsive to IVMP. A dose-analysis found that there was no difference between the higher (2000 mg) and lower (500 mg) dosage of Rituximab on clinical response [100]. A recent retrospective cohort study has corroborated this finding that using lower doses of Rituximab, a single infusion of 100 mg, yielded efficacious results, reducing the likelihood of AE [101].

The conflicting outcomes between the 2 RCTs may be due differences in subject recruitment. The Mayo clinic cohort had a significantly longer TED disease duration (mean 30 months), compared to 4.5 months in the Milan study and that Rituximab maybe more effective in patients with acute inflammatory TED than in more chronic disease [102]. Other sources of bias may have impacted the results. The Milan study was terminated early due to frequent TED reactivation in the IVMP arm. In addition, the Rituximab infusion protocol was altered following the first 12 participants [100]. The Mayo clinic study concluded prior to reaching the full quota of participants due to challenges faced recruiting patients [99].

A recent meta-analysis evaluating the use of Rituximab in TED from 2021, evaluated 12 cohort or RCTs (152 patients) and concluded that Rituximab had a rapid and long-lasting impact on the reduction of both CAS and TRAb [103]. Nevertheless the small and limited number of RCTs, impacts the ability to effectively evaluate the efficacy of Rituximab in patients with TED. This conclusion was also supported in a 2022 Cochrane review that further larger multi-centre studies are required to ascertain the therapeutic benefit of Rituximab in TED [104].

### Teprotumumab

Teprotumumab, an Insulin Growth Factor Receptor 1 (IGFR-1) partial antagonist was originally developed unsuccessfully as an oncology drug to treat solid tumours [105]. It was subsequently repurposed as a therapeutic agent for TED to target the IGFR-1 receptor co-localised with the TSHR on orbital fibroblasts [14–16]. Activation of IGF-1 leads to an intra-cellular cascade producing many of the typical signs and symptoms associated with TED [19, 20].

The Phase 2 and 3 OPTIC trials evaluated the use of Teprotumumab versus placebo in active moderate-to severe disease. Both studies had similar protocols and methodologies with treatment given every three weeks intravenously for 24 weeks [106, 107]. In the Phase 2 trial (*n* = 88), the primary end point was at Week 24 and defined as a ≥2 point reduction in CAS and ≥2 mm of proptosis. This occurred in 69% of patients on Teprotumumab compared to 20% on placebo (*P* < 0.001) with 43% of the treatment cohort responding at week 6 compared to 4% on placebo (*P* < 0.001).

The mean reduction from baseline of CAS was 3.43 mm versus 1.85 mm in placebo (*P* < 0.001). Proptosis also showed promising results with a mean reduction of 2.46 mm in the treated group compared to 0.15 mm in placebo (*P* < 0.001). Of those patients who received the drug, 40% had a reduction of proptosis of ≥4 mm versus 0% on placebo. Additionally, treated patients showed significant positive outcomes in diplopia and GO-QOL [106].

The subsequent prospective double‐blinded RCT (OPTIC *n* = 83), had a primary outcome assessing proptosis alone measured at 24 weeks. A positive response was defined as a reduction in proptosis of ≥2 mm. The study found 83% of patients on treatment had a positive response versus 10% on placebo (*P* < 0.001). Secondary outcomes evaluating overall response including a reduction in CAS, diplopia and GO-QOL were also better in the treatment arm (*P* ≤ 0.001). Six teprotumumab treated patients, demonstrated reduced extraocular muscle, orbital fat volume or both on MRI [107].

The US Food and Drugs Administration (FDA) and more recently the Brazilian Health Regulatory Agency, approved Teprotumumab for any level of severity, activity and duration of TED [108]. However, there is a question as to how durable the effects of Teprotumumab are. A study performed by the original investigators, reviewed the long-term outcomes of patients from the original studies using follow-up data. The subsequent analysis, which utilised pooled data, found patients, from both trials, responding to treatment with proptosis had fallen to 67% at 72 weeks [109]. A subsequent RCT re-treatment trial (OPTIC-X) was performed on patients deemed non-responders from the original OPTIC study or those who flared on follow-up. The trial consisted of patients who would be treated for the first time i.e who were previous placebo patients (*n* = 37) or those who were formerly in the Teprotumumab arm requiring re-treatment (*n* = 14). Both groups achieved a response. 89.2% of the former placebo group achieved an average reduction in proptosis of 3.5 mm, comparable to the original OPTIC study. Of those previously treated with Teprotumumab, five patients were original non-responders and eight were previous responders who experienced a flare. Only two patients, in the original non-responder group, responded when re-treated, two patients had to discontinue treatment early and one patient had a partial response. Of the eight original responders who experienced a flare, 62.5% responded on re-treatment [110]. The study suggests there may be a subset of TED patients who are not responsive to Teprotumumab. There may be a gradually diminishing effect of the treatment over time, particularly in a sub-group of patients where re-infusion is required. Further studies are required to evaluate the longevity of the therapeutic response further.

Improvements in CAS were noted with an overall treatment difference of 60% between intervention and placebo (*p* < 0.0001). Significance was also recorded at each time point. Diplopia scoring had a similar improvement with a ss treatment difference of 39% (*p* < 0.0001). Although in a subgroup analysis this was not the case for tobacco users (*p* = 0.086). Finally, GoQoL scores were significantly higher at week 24 in the intervention arm versus placebo for both visual function and appearance [110].

For AE, the studies showed the majority of patients suffered only mild or moderate events. These mild-to-moderate events consisted primarily of fatigue, muscle spasms, hair loss, diarrhoea and nausea. Hyperglycaemia, particularly in patients who had a history of diabetes, was observed. This is unsurprising considering the role of IGF-1 in the regulation of blood sugar levels. Patients often responded to medical management when this AE was identified [106, 107]. New cases of inflammatory bowel disease have been described and a case of amyloid encephalopathy which responded to plasmapheresis [111, 112].

Sensorineural-hearing loss, has been recorded in patients with variability in severity. The mechanism is not fully understood, with some patients self-resolving whilst others suffering permanent hearing-loss [113]. In the phase II trial, approximately 7% of patients suffered some form of hearing-loss or tinnitus with a degree of heterogeneity of onset, duration and symptomology. The majority of these were self-resolving [18]. The phase III OPTIC trial, reported hearing impairment in five patients <5% in the trial group with a significant degree of variability ranging from tinnitus to deafness all of which resolved [107]. The OPTIC-X study corroborated these findings with four patients identified as having mild hearing related AE [110]. All of these patients resolved with the exception of one patient whose tinnitus was present at the last visit with muscle spasms. The patient’s treatment was discontinued following the sixth infusion [110].

However, a prospective observational case series investigating hearing related AE in patients infused with Teprotumumab for TED found a higher rate of hearing impairment [114]. Of the 27 patients infused, 81.5% developed some new auditory symptomology after a mean of 3.8 infusions. Although the majority of patient’s symptoms self-resolved, six patients had persistent hearing loss. All of these patients had reduced auditory thresholds determined on audiometric testing with four requiring long-term hearing aids. Further studies are required to fully ascertain the AE profile and long-term prognosis [114].

Teprotumumab is the first treatment to have successfully achieved medical orbital decompression with large improvements in proptosis. Whilst the drug is unlikely to replace surgical orbital decompression in its entirety, its impact has been significant leading to an explosion of interest in the development of new TED therapeutics.

## Development of future therapies

The recent consensus statement by The American and European Thyroid Associations, have outlined the future direction of TED management. This will involve a greater degree of personalisation utilising activity and severity scores as well as disease phenotype [25]. Several future therapies are adopting this approach which we will explore in the section below.

## Targeting orbital fibroblast receptors

### Biosimilar & small molecule IGFR-1 receptor blockers and antibodies

A number of biosimilar MABs to IGF-1 are in development including VRDN-001, VRDN-002, IBI311 and ZB001 each at various trial stages. A report on the interim safety results of a phase1/2 trial evaluating the safety and efficacy of VRDN-001 (NCT05176639) found the antibody was well tolerated in the 13 patients assessed with no reported hyperglycaemia or hearing loss at 50 days [115]. Another antibody to IGF-1, VRDN-002, is a second-generation antibody based on VRDN-001 but with the Fc segment modified to allow for a longer half-life and thus more convenient dosing. A study was performed evaluating its pharmacokinetics on cynomolgus monkeys, showing a positive AE profile with high bioavailability when given subcutaneously with a prolonged half-life [116]. IBI311, is currently in phase III trials (NCT05480579) assessing the impact on proptosis at 24 weeks in Chinese patients with TED. ZB001, is currently recruiting for phase 1/2 trials evaluating a similar patient cohort (NCT05176639).

Teprotumumab is a large molecular compound required to be given via intravenous infusion [110]. A current Phase IV post-marketing study is being conducted to evaluate the optimal dosing regimen. Intravenous infusion, provides challenges including patient inconvenience, a costly set-up with specialised staff and careful monitoring required. Alternative routes of administration, such as oral delivery, would remove many of these barriers. A drug currently under investigation is Linsitinib a selective small-molecule dual inhibitor. Due to its prior evaluation as an oncology product, the drug is understood to be well tolerated. It acts via the tyrosine kinase signalling pathway inhibiting both the IGF-1R and Insulin Receptor [117]. An in-vivo study with mice assessed the impact of Linsitinib at different points on Rundle’s curve. The drug was initiated either in the early active or late inactive phase orally for four weeks. In the Linsitinib group, hyperthyroidism was prevented early in the disease. Late in the disease, Linsitinib significantly reduced immune infiltration of T-cells and macrophages into the orbit. There was also normalisation of brown adipose tissue, a reduction in muscle oedema and inflammation confirmed by functional MRI [118]. These findings have led to the development of a phase 2b clinical trial (LIDS study). The study is actively recruiting and aims to evaluate Linsitinib in active moderate-to-severe TED (NCT05276063).

### TSH-R blockers

TSH-Receptors are overexpressed within orbital tissue in TED patients and when activated results in adipogenesis within the orbit [11–13]. IGFR-1 and TSH-Receptors, are also co-localised on orbital fibroblasts, and thought to have a synergistic relationship with a degree of cross-communication. Subsequently, activation of one receptor may activate the intracellular cascade of the other [19, 20]. The role of the TSH-Receptor antibodies, is unclear although they likely play a crucial role in the pathogenesis of TED. A variety of antibodies exist including stimulating, blocking and neutralising and how these antibodies interact with the TSH-Receptor at different type points on Rundle’s curve is not fully understood [119, 120]. The different forms of antibodies, may predominate and impact certain ethnic populations to various degrees [121, 122].

K1-70 is a recombinant human IgG immunoglobulin targeted at the TSH-Receptor preventing stimulation and ligand binding [123]. Phase I trial data found the drug, administered IV or IM, was well tolerated with no serious AE. Six patients reported symptomatic improvements in their GO following infusion and five had an objective improvement in proptosis from baseline [124]. The use of K1-70 in a single case report, evaluated its effects on a patient with TED and Follicular Thyroid Cancer (FTC), showing a decline in TSAb, CAS and regression or stabilisation of FTC [125].

Small molecule TSH-R blockers are under development and confer similar advantages outlined for small molecule IGFR-1 Blockers. The mechanism often differs from the traditional TSH-R antagonist, in that they are not typical competitive antagonists, but interfere with signal transduction [126, 127] The S37a molecule is particularly promising as it is highly specific to the TSHR [126, 128]. Its several chiral centres create a rigid scaffold that prevents the molecule acting on receptors with similar homology e.g. Luteinising Hormone Receptor even at high concentrations, conferring a more limited AE profile [126]. Human studies have not yet been performed but the molecule showed excellent tolerability during in-vivo studies [128]. Next generation molecules such ANTAG3, which show similar benefits to S37a, but with the added benefit of inhibiting hyaluronic acid secretion at maximal effective doses are under development [127, 129].

Dual blockage of the IGFR-1 and TSHR may interrupt synergistic cross-communication preventing activation of the intracellular cascade [19, 20]. In-vitro studies with Linsitinib (IGFR-1 blockade) in conjunction with ANTAG3 (TSH-R blockade) showed enhanced inhibition of hyaluronan production [126, 127].

## Targeting components of the inflammatory cascade

### Cytokines

Several therapeutic agents, targeting cytokines within the TED inflammatory cascade, are under evaluation. Vunakizumab, also known as SHR-1314, is a MAB that targets the IL-17a receptor [130]. Th17-cells, a sub-set of CD4+ T-cells, are involved in several autoimmune diseases including RA and Lupus [131]. Mature Th-17 cells secrete IL-17A, a pro-inflammatory cytokine, shown to propagate inflammation, fibrosis and adipogenesis via orbital fibroblasts in TED [132].

Furthermore, patients found to have single nucleotide polymorphisms (SNPs) of IL-17a have a higher susceptibility to develop TED [133]. Vunakizumab, is also under investigation for the treatment of Psoriasis and recently published phase I trial data shows good tolerability [130]. A phase II study investigating the safety and efficacy of the drug in Moderate-to-Severe active TED patients is currently underway (NCT05394857). Another IL-17a blocker, Secukimumab, is currently undergoing a double-blinded phase III RCT (ORBIT) in TED patients with active moderate-to severe disease (NCT04737330).

LASN01, is being trialled for a number of pro-fibrotic conditions including TED, pulmonary fibrosis and systemic sclerosis [134]. It targets the IL-11 receptor which is thought to play a role in fibrotic tissue remodelling although the evidence base in TED is not as well established. A recent study showed that IL-11 levels were overexpressed in TED patients versus controls and positively correlated to CAS. The authors found that IL-11 may be implicated in a pro-fibrotic phenotype switch of orbital fibroblasts in TED [135]. A phase I study is recruiting to assess the safety, tolerability and pharmacokinetics of LASN01 in patients with TED and Pulmonary fibrosis (NCT05331300).

### T-cells

Otelixizumab, also identified as TRX4, is a MAB targeting the CD3 receptor, resulting in apoptosis or anergy of activated effector T-cells. It was previously developed to treat Type 1 Diabetes Mellitus and was well tolerated with limited and transitory AEs [136]. Previous studies have shown orbital CD3+ T-cell infiltration being correlated with CAS and disease duration in TED [137]. Furthermore, following the introduction of the drug, CD3+ T-Cells have been shown to decline by 40% in the peripheral blood [136]. A phase II trial to evaluate the safety and pharmacokinetics of TED patients being treated with Otelixizumab was terminated prematurely. The reason given, was the need for further studies to determine the most efficacious dose of Otelixizumab. Only two patients were recruited prior to termination of the study (NCT01114503).

### B-cells

Belimumab, is a well-established drug in the treatment of lupus [138]. It is a recombinant, human MAB directed at the soluble B-lymphocyte stimulator protein (BLyS) also known as BAFF [139]. BLyS, is a cytokine, that is integral to the differentiation and activation of B-Cells to plasma cells. Plasma cells may secrete pathogenic immunoglobulins, such as TSH-Receptor antibodies, which is key in the pathogenesis of TED. In addition, serum BLyS levels have been shown to be higher in patients with TED relative to controls [140]. Previous phase I studies, in the treatment of systemic lupus erythematosus (SLE), have established Belimumab’s safety profile [139, 141]. An ongoing single blind RCT evaluating Belimumab versus IVMP in patients with moderate-to-severe active TED reported its interim results. Belimumab was as effective as Methylprednisolone in the inactivation of TED with success in 90% of patients at 24 weeks (mean CAS 1.5 and 1.33 respectively) although it was slower in onset [142]. Diplopia scores and proptosis were not ss. Belimumab was better tolerated than IV MP. It may show promise as an alternative treatment to IVMP when contraindicated or poorly tolerated [142].

### Intracellular recycling

The neonatal fragment crystallizable receptor (FcRn) is key in prolonging the half-life and preventing the degradation of IgG antibodies in circulation via a recycling mechanism [143, 144]. Batoclimab (RVT-1401), a fully MAB, blocks this function by competitively adhering to the IgG receptor site on FcRn. Subsequently there is enhanced catabolism of IgG resulting in a drop in plasma levels [145]. The drug is undergoing phase II trials for Myasthenia Gravis and currently utilised in Neuromyelitis Optica. In TED it may decrease circulating TSHR and IGF-1 antibodies [146, 147].

A double-blind RCT evaluated the 12 weeks change in proptosis of Batoclimab compared to placebo in active moderate-to severe TED patients [144]. The trial was prematurely terminated due to an unexpected rise in serum cholesterol in the treatment arm. As a consequence, data from approximately 16% of the recruited patients were not analysed and the conclusion did not find a ss difference at 12 weeks between Batoclimab and placebo on improving proptosis. Although there was a ss muscle volume reduction (*P* < 0.03) measured on Computerised Tomography. Nevertheless, the study showed good tolerability of Batoclimab with reversal of drug induced hypercholesterolemia and hypoalbuminemia within eight weeks of discontinuation. Preliminary data from a healthy volunteer study suggest co-treatment with a statin can prevent a rise in LDL levels [148]. A second proof-of-concept study found a ss decline in anti-TSHR antibody levels and total IgG serum levels (*P* < 0.001) in subjects taking Batoclimab [149]. An upcoming phase III RCT comparing Batoclimab to placebo over a 24-week period is under recruitment (NCT05517421 and NCT05524571).

### Other therapies

Several other immunomodulatory therapeutics are being assessed including Iscalimab (anti-CD40 MAB) and Fingolimod (sphingosine-1-phosphate receptor modulator) [150, 151]. A new and developing area of investigation is the impact of the microbiome on TED disease activity and severity [152].

## Conclusion

Successful TED therapy reverses visual dysfunction, disfigurement and improves patient QoL with good safety and tolerability.

Previous options for medical treatment were restricted to optimising thyroid endocrine treatment and using non-specific immunosuppression. Elucidation of the molecular pathophysiology has provided a variety of immunological targets for new therapeutic developments, likely to increase our arsenal. However, in a new age of personalised medicine, the challenge will lie in identifying the most effective target for each patient rather than any single agent being a panacea.

Long-term safety profiles require clarification as there may be a ‘Pandora’s Box’ of unintended immunological consequence downstream as seen in other diseases. Moreover, deploying these advances in the ‘real world’ may entail significant expense and lead to a multi-tiered divergence and availability of treatment modalities between healthcare systems due to funding disparities.

## Summary

### What is known about this topic

- TED has a significant disease with a population prevalence of 2% and an annual incidence of 20 cases/100,000 persons. Consequential complications can include blindness and disfigurement, and it exacts a significant economic and psychosocial burden.
- TED involves a complex pathophysiology which includes interactions between cellular and humoral-based autoimmune dysfunction.
- A multitude of therapeutics have been utilised to impact both the activity and severity of disease. Utilisation of which therapeutics are utilised often varies on locality and clinician confidence.

### What this study adds

- Previous therapies have been broad-based, acting as a blunt instrument on the mechanism of TED with various degrees of success but often accompanied by a significant side effect profile.
- Developments in targeted therapy of the molecular pathway of TED, spearheaded by Teprotumumab, have led to an array of treatments optimising impact whilst possibly minimising their side effect profile.
- Long-term safety profiles will require clarification of these novel therapeutics as unintended 109 immunological consequences downstream may become manifest as seen in other diseases.
